# Toward a Better Understanding of the Relationship between Belief in the Paranormal and Statistical Bias: The Potential Role of Schizotypy

**DOI:** 10.3389/fpsyg.2016.01045

**Published:** 2016-07-14

**Authors:** Neil Dagnall, Andrew Denovan, Kenneth Drinkwater, Andrew Parker, Peter Clough

**Affiliations:** Manchester Metropolitan UniversityManchester, UK

**Keywords:** schizotypy, paranormal belief, heuristical bias, perception of randomness, conjunction fallacy

## Abstract

The present paper examined relationships between schizotypy (measured by the Oxford-Liverpool Inventory of Feelings and Experience; O-LIFE scale brief), belief in the paranormal (assessed via the Revised Paranormal Belief Scale; RPBS) and proneness to statistical bias (i.e., perception of randomness and susceptibility to conjunction fallacy). Participants were 254 volunteers recruited via convenience sampling. Probabilistic reasoning problems appeared framed within both standard and paranormal contexts. Analysis revealed positive correlations between the Unusual Experience (UnExp) subscale of O-LIFE and paranormal belief measures [RPBS full scale, traditional paranormal beliefs (TPB) and new age philosophy]. Performance on standard problems correlated negatively with UnExp and belief in the paranormal (particularly the TPB dimension of the RPBS). Consideration of specific problem types revealed that perception of randomness associated more strongly with belief in the paranormal than conjunction; both problem types related similarly to UnExp. Structural equation modeling specified that belief in the paranormal mediated the indirect relationship between UnExp and statistical bias. For problems presented in a paranormal context a framing effect occurred. Whilst UnExp correlated positively with conjunction proneness (controlling for perception of randomness), there was no association between UnExp and perception of randomness (controlling for conjunction).

## Introduction

Heuristics are simple mental rules or shortcuts, which ease cognitive load and facilitate rapid formation of judgments and decisions (Tversky and Kahneman, 1974). While heuristics typical yield reasonable judgments, they can also produce systematic deviations from logic and probability (Costello and Mathison, 2014). Recent research indicates that belief in the paranormal is associated with susceptibility to heuristic bias, particularly misrepresentation of chance (Dagnall et al., 2007, 2014, 2016) and conjunction fallacy (Rogers et al., 2009, 2011, 2016). The present paper examined the degree to which level of schizotypy influenced this relationship. This was a logical extension to previous research because schizotypy correlates with belief in the paranormal and is associated with proneness to reasoning and cognitive bias. For instance, jumping to conclusions (Moritz and Woodward, 2005; Sellen et al., 2005) and tendency to discount disconfirmatory evidence (Buchy et al., 2007).

Schizotypy is a rich and complex psychopathology concept (Lenzenweger, 2015). Researchers use schizotypy as a tool for investigating schizophrenia and psychosis associated phenomena because schizotypy measures assess non-clinical populations and thus avoid confounds associated with schizophrenic patients (i.e., symptom severity and general decline in cognitive performance) (Claridge, 1988; Yoon et al., 2008). The relationship between schizotypy and schizophrenia, however, is not a simple one. Different views exist on the continuum between schizotypy, mental health and mental illness (i.e., quasi-dimensional, dimensional and fully dimensional). The present paper restricts itself to the notion that schizotypy is a personality trait (Claridge and Beech, 1995) assessed on a continuum, ranging from relative psychological health to schizophrenia (psychosis) (Barrantes-Vidal et al., 2015). This perspective compliments the observations that schizotypy presents within the general population and does not necessarily result in psychopathology. Indeed, non-clinical community respondents can obtain high schizotypy scores without exhibiting schizophrenia spectrum symptoms (Dembińska-Krajewska and Rybakowski, 2014).

The inclusion of schizotypy is a logical extension to previous work for several reasons. Firstly, there is a well-documented positive correlation between schizotypy and belief in the paranormal (Hergovich et al., 2008). Hergovich et al. (2008) found that schizotypy predicted key facets of belief in the paranormal (i.e., precognition, psi, witchcraft and spiritualism). This association arises in part from construct overlap. One of the Schizotypal Personality Disorder diagnostic criteria refers specifically to odd beliefs or magical thinking (Hergovich et al., 2008). This relationship suggests that schizotypy may also influence correlates of paranormal belief. Secondly, the directional nature of the correlation between schizotypy and paranormal belief is uncertain. Specifically, it is unclear whether belief in the paranormal produces high schizotypy scores, schizotypy promotes paranormal beliefs (Hergovich et al., 2008), or the relationship is explained by a common third variable.

The association is complicated further because schizotypy and belief in the paranormal interact differently with related beliefs. For example, paranormal belief (vs. level of schizotypy) better predicts traditional religious contents, superstitious thoughts and belief in the existence of extraordinary life forms (Hergovich et al., 2008).

Finally, previous work indicates that belief in the paranormal and schizotypy influence perception of causal relationships. Principally, perception of illusory causality and proneness to connectedness (the tendency to perceive co-occurring events as meaningfully associated). Perception of randomness in turn is likely to affect appreciation of chance (randomness) and susceptibility to heuristical bias. Thus, the present study tested the previously unassessed assertion that schizotypy effects susceptibility to heuristic bias.

Paranormal belief is moderately associated with probability misjudgment (Blackmore and Troscianko, 1985; Bressan, 2002; Dagnall et al., 2007, 2014). A seminal example is Blackmore and Troscianko (1985), who reported that believers in psi (vs. non-believers) performed less well on probability judgment tasks. Psi refers to the unknown factor in extrasensory perception and psychokinesis experiences. Subsequent research produced similar findings, but focused on a limited number of reasoning problem types. Observing this, Dagnall et al. (2007) conducted a study including a range of problem-solving tasks. Items evaluated four key reasoning domains: perception of randomness (judging the likelihood of obtaining strings/sequences), base rate (probability of a stated outcome in relation to presented information), conjunction fallacy (determining whether co-occurring events were more likely to occur than single, constituent events) and derivation of expected value (evaluating odds in order to maximize pay-outs). Performance on perception of randomness tasks emerged as the best predictor of level of paranormal belief. This finding supported the notion that believers in the paranormal possess a tendency to perceive random events (coincidences) as causally related (meaningful) (Brugger and Taylor, 2003).

Relatedly, Rogers (Rogers et al., 2009, 2011) reported a positive correlation between susceptibility to conjunction fallacy and belief in the paranormal. Conjunction fallacy refers to instances where event co-occurrence [P(A&B)] (conjunction), is rated more likely than constituent events, P(A) or P(B) (Tversky and Kahneman, 1983; Hertwig and Chase, 1998). The work of Rogers is important because few previous studies considered the role conjunction plays in the development and maintenance of paranormal beliefs.

Using a specially designed Scenario Judgments Questionnaire (SJQ), (Rogers et al., 2009) found that paranormal believers (vs. non-believers) made more conjunction errors and that context effected susceptibility to conjunction; standard problems produced more errors than those presented in a paranormal framework (Rogers et al., 2009). The SJQ is a measure comprised of 16 conjunction vignettes divided into non-paranormal/conventional (e.g., queuing for airport coffee) and paranormal (e.g., alleged precognition) events. Rogers et al. (2011) reproduce the finding that believers (vs. non-believers) produced more conjunction errors, but failed to replicate the outcome that performance varied as a function of problem type (conventional vs. paranormal).

Conjunction effects are typical weak and significant outcomes reported inconsistently across the literature. Noting this, Dagnall et al. (2014) investigated further the degree to which specific probabilistic biases (perception of randomness, base rate, conjunction fallacy and probability) were associated with belief in the paranormal and proneness to reality testing (RT) deficits (Inventory of Personality Organization-Reality Testing; Lenzenweger et al., 2001). To ensure results were not an artifact of the paranormal scale used the study employed a range of measures (Manchester Metropolitan University-New, MMU-N, Dagnall et al., 2010a,b; Revised Paranormal Belief Scale, RPBS, Tobacyk and Milford, 1983; Tobacyk, 2004; and Australian Sheep–Goat Scale., ASGS, Thalbourne and Delin, 1993).

Multiple regression revealed the best predictor of belief in the paranormal and proneness to RT deficits was perception of randomness. Performance on conventional conjunctions correlated only with the Tradition Paranormal Beliefs (TPB) dimension of the RPBS. Paranormal conjunctions correlated with both paranormal belief and proneness to RT deficits. Overall findings indicated that conjunction was not strongly associated with belief in the paranormal.

Considering these findings, Rogers (2014) in the context of general paranormal belief concluded that conjunction represents a particular instance of misrepresentation of chance (see Dagnall et al., 2016). This notion is consistent with the small reported effect sizes and the observation that conjunction adds no/little unique variance to regression models including perception of randomness (Dagnall et al., 2014). Cumulatively, results suggest that susceptibility to conjunction indirectly indexes perception of randomness. Findings agree with Bressan’s (2002) general proposition that believers in the paranormal possess a lower subjective chance threshold, which inclines them to perceive unrelated events as causally related.

Schizotypy also influences perceptions of causality and connectedness. Specifically, illusory causation is associated with symptoms related to positive schizotypy, principally magical ideation (Rominger et al., 2011). Within high schizotypes this manifests as a tendency to see meaning within random patterns (Gianotti et al., 2001; Farias et al., 2005). A specific example is the propensity to view coincidences as causally related (Diaconis and Mosteller, 1989). More generally, susceptibility to illusory causality correlates with scores on cognitive-perceptual measures, such as delusional ideation (Bressan, 2002). In the context of belief in the paranormal, illusory causation may manifest as the tendency to perceive random events as causally related.

The relationship between schizotypy and illusory causation, however, is not a simple one (Fyfe et al., 2008). Fyfe et al. (2008) examined the association between schizotypy and propensity to see connections between unrelated stimuli or events (apophenia) and found a positive association on only *one* of a series of tasks (triangles). Schizotypy failed to correlate significantly with performance on animated contingency and stories tasks. Consequently, Fyfe et al. (2008) concluded that within healthy individuals, the tendency to perceive connectedness represents a weak effect, apparent only under certain conditions (i.e., situations where high ambiguity exists).

Rominger et al. (2011) explains illusory causation in terms of loose cognitive control. Specifically, broader or less rigid associative networks and weaker inhibition of irrelevant memory content. Poorer appreciation of chance may also be associated with allusive thinking or looseness of associations (Rominger et al., 2011). Studies report individuals high in positive schizotypal symptoms (vs. low) perceive more meaning within random patterns and generate a higher number of unusual words (Gianotti et al., 2001; Farias et al., 2005). In addition, individuals high in delusional ideation seek less information prior to reaching a decision. This expresses as the inclination to jump to conclusions, where hypotheses related to decision-making process are accepted or rejected prematurely.

Within delusional individuals, the propensity to make inaccurate judgments based on insufficient information may contribute to delusion formation and maintenance (Garety and Freeman, 1999). Collectively, studies indicate that schizotypy is associated with inappropriate attribution of causation and misperception of randomness (factors linked also with belief in the paranormal).

Whilst belief in the paranormal and schizotypy are both associated with misperception of chance and illusory causation, their relative contribution to heuristic bias requires exposition. A useful starting point is the work of Williams and Irwin (1991), who delineated paranormal belief systems as a rational (if deviant) attempt to achieve a metacognitive understanding of the world, is useful. Williams and Irwin (1991) proposed that belief in the paranormal represents believers’ subjective efforts to structure the world in terms of magical notions of causation. Paranormal beliefs act as a framework for structuring odd beliefs and/or magical thinking (schizotypal related cognitions). Believers perform poorly on tasks related to perception of randomness because they engage in a sophisticated form of internal reasoning founded on magical ideation. This represents an individual worldview based upon a subjective preferential thinking style, rather than a defective understanding of probability.

This rational view contrasts with the conventional interpretation, which attributes endorsement of paranormal beliefs within schizotypes to impaired psychological functioning (cognitive, perceptual and affective distortions); full-blown schizophrenic symptoms encompass the tendency to embrace paranormal attributions alongside a lack of self-awareness. Within sub-clinical populations, explanations based upon thinking style preference are more apposite, given the prosaic nature of paranormal beliefs.

### The Present Study

This paper tested a series of predictions examining relationships between schizotypy, belief in the paranormal and statistical bias. Hypotheses considered the dimensional structural of schizotypy and the factorial composition of paranormal belief in order to assess which sub-measures were most strongly associated with general and specific (misrepresentation of chance and conjunction fallacy) propensity to probabilistic reasoning bias. Particularly, the study examined the degree to which belief in the paranormal mediated the relationship between schizotypy and statistical bias.

The O-LIFE assessed level of schizotypy. The O-LIFE developed from work on the personality approach to measuring schizotypy, whilst other scales, such as the Schizotypal Personality Questionnaire-Brief (Raine and Benishay, 1995), derive from clinical work based on clinical measurement tools. For this reason and because the target sample was normal healthy individuals the O-LIFE was considered most appropriate (Mason, 2015).

#### Schizotypy and Belief in the Paranormal

Acknowledging preceding work, the researchers hypothesized that the Unusual Experiences (UnExp) dimension of the O-LIFE scale would best predict belief in the paranormal. The UnExp subscale assesses positive symptoms of psychosis and contains items measuring susceptibility to perceptual aberrations, magical thinking and hallucinations. Prevalence of these characteristics is likely to foster cognitions/perceptions conducive to the formation of paranormal beliefs (i.e., proneness to experiencing strange perceptual and cognitive sensations and/or magical interpretations of occurring events) (Dagnall et al., 2010; Dembińska-Krajewska and Rybakowski, 2014).

Other O-LIFE dimensions [cognitive disorganization, impulsive non-conformity (ImpNon) and introverted anhedonia] relate less obviously to anomalous beliefs. Indeed, cognitive disorganization (unconventional trains of thought; *disorganized thinking*) and ImpNon (the failure to follow social rules non-compliance with conventional or established social roles; *lack of self-control*) only indirectly index elements of anomalous thinking/beliefs. Accordingly, significant but weak relationships were expected.

Regarding introverted anhedonia (blunted affect, antisocial behavior and lack of ability to feel pleasure; *negative schizotypy*), previous research evinces that negative aspects of schizotypy are not primarily involved with the development of paranormal beliefs. Instead, negative schizotypy plays a key role in the affective interpretation of subjectively perceived paranormal experiences (Irwin and Green, 1998; Schofield and Claridge, 2007). Hence, no significant association was anticipated between paranormal belief and introverted anhedonia (Irwin and Green, 1998).

Consistent with Williams and Irwin (1991), the authors hypothesized that belief in the paranormal would structure cognitions and perceptions related to UnExp/magical ideation and consequently, increase the tendency to perceive non-causally events as related. Thus, whilst scores on high schizotypy (particularly, UnExp) correlate negatively with tasks assessing statistical bias, this relationship would be mediated by level of paranormal belief. Operationally, this manifests as decreased performance on tasks indexing perception of chance (particularly, those assessing perception of randomness). The notion that misrepresentation of chance is more strongly related to belief in the paranormal corresponds with previous research (Dagnall et al., 2014, 2016; Rogers, 2014) and Arnott’s (1998, 2006) taxonomy of decision biases, which arose as a response to inadequacies within previous classification systems (e.g., Tversky and Kahneman, 1974).

Within the taxonomy the statistical bias category, embraces chance, mistaking random events for essential process characteristics (Wagenaar, 1988) and conjunction (the overestimation of probability in compound problems; Tversky and Kahneman, 1983). The statistical bias category is central to the present paper because it provides a rationale for why propensity to conjunction fallacy is less strongly associated with belief in the paranormal. This occurs because conjunction fallacy represents a specific instance of misrepresentation of chance (Dagnall et al., 2014, 2016; Rogers, 2014). Thus, whilst misrepresentation of chance and proneness to conjunction correlate positively, conjunction contributes little unique variance. Although, predictions concerning relationships between schizotypy, belief in the paranormal, and statistical bias arose from careful consideration of previous research, no prior work has investigated the inter-relationship between these factors.

A further consideration was the fact that previous research reports that the relationship between belief in the paranormal and statistical bias varies as a function of belief type (Dagnall et al., 2014, 2016). Poorer statistical performance is more strongly associated with Traditional Paranormal Beliefs (TPB) than New Age Philosophy (NAP). These dimensions reflect the differential functioning of beliefs (individual vs. social) (Lange et al., 2000). NAP (psi, witchcraft, spiritualism and astrology) instills control over external events at the *individual/personal level* (Irwin, 1992), whilst TPBs (traditional religious beliefs, witchcraft and precognition) regulates external events at a *social cultural level* (Goode, 2000).

Preceding studies note that these functional differences influence susceptibility to heuristic bias. Particularly, Dagnall et al. (2014) in line with previous work (Hergovich et al., 2008; Wilson, 2013) observed that whilst perception of randomness correlated weakly with both NAP and TPBs, conjunction correlated only with TPBs. Thus, TPBs should more strongly relate to statistical bias than NAP, and the effect should be stronger for misperception of randomness than proneness to conjunction. Hence, the authors tentatively predicted that TPBs would have a stronger mediating effect than NAP.

Finally, this study included paranormal problem types alongside standard problem types. These index the degree to which participants endorse paranormal explanations in preference to optimal statistical solutions. The researchers anticipated that performance on paranormal problems would correlate negatively with schizotypy.

## Materials and Methods

### Participants

A sample of 254 participants (69 male, 27% and 185 female, 73%) took part in the study. Mean age 26.66, *SD* = 9.81, range 18–71 years. Males, *M* = 28.84, *SD* = 10.52, range 20–65 years, and females, *M* = 25.84, *SD* = 9.43, range 18–71 years. Participant recruitment was via emails to university staff and students and local stakeholders (businesses, leisure and vocational/sports classes). The sample comprised 60% enrolled undergraduate students (59% Psychology, 30% Health Care, and 11% Arts and Humanities) and 40% non-students. Prior to participation, a question asked whether participants had previously studied heuristic bias. If participants endorsed the question, participation discontinued.

### Measures

#### Probabilistic Reasoning Tasks

Four problem types derived from Dagnall et al. (2007, 2014) assessed probabilistic reasoning: perception of randomness, conjunction fallacy, paranormal perception of randomness and paranormal conjunction fallacy. Items were organized into five counter-balanced sections, which contained one of each problem type.

After reading each problem, participants indicated the most probable outcome from a range of alternatives.

#### Perception of Randomness

These problems evaluated participant’s ability to judge the likelihood of strings/sequences (e.g., ‘imagine a coin was tossed six times. Which pattern of results do you think is most likely?: (a) HHHHHH, (b) HHHTTT, (c) HTHHTT, (d) all equally likely’).

#### Conjunction Fallacy

Participants selected the most probable outcome from presented statements. Alternatives included single and co-occurring events [e.g., ‘two football teams (Team A and Team B) are playing in a local derby. What is the most likely outcome of the game?: (a) Team A scores first, (b) Team A scores first and win, (c) Team A scores first and loses, (d) Team A scores first and the game is drawn’].

#### Paranormal Perception of Randomness

Items possessed the same underlying structure as standard perception of randomness problems. The only differences being that judgments about the likelihood of strings/sequences occurred within a paranormal context. For example, ‘A famous psychic, with renowned paranormal abilities, has successfully predicted the outcome of the last six annually held boat races between two famous English Universities’ [University A and University B]. This year the psychic predicts University B will win. Which of the following is most likely?: (a) University A will win the event, (b) University B will win the event, (c) University A and University B are both equally as likely to win the event.

#### Paranormal Conjunction Fallacy

Similarly, paranormal conjunctions contextualized conjunctions within a paranormal setting. Problems possessed the same underlying structure as standard conjunctions; event intersection probability could not exceed single (constituent) event likelihood (*cf*. Tversky and Kahneman, 1982, 1983). For instance, ‘Andrew often sits by the telephone at work. Just as he is thinking about his friend (Elaine), she rings: (a) Elaine rang because Andrew was thinking about her [event intersection], (b) Andrew was thinking about Elaine because she was about to ring [event intersection], (c) Elaine rang [single event].’

#### The Oxford-Liverpool Inventory of Feelings and Experiences (O-LIFE)

The O-LIFE brief is shortened version of the original 104-item scale (Mason et al., 1995). The O-LIFE measures schizotypal personality traits in non-clinical individuals. The O-LIFE brief contains 43 items divided into four sub-scales: UnExp, Cognitive Disorganization (CogDis), Introvertive Anhedonia (IntAn) and ImpNon (Mason et al., 2005). The UnExps scale contains 12 items phenomenologically related to the positive symptoms of psychosis and thus assesses positive schizotypy (perceptual aberrations, magical thinking and hallucinations). The CogDis sub-scale comprises 11 items reflecting thought disorder and other disorganized aspects of psychosis. Particularly, items tap poor attention/concentration, poor decision-making and social anxiety. The IntAn sub-scale is composed of 10 items measuring negative schizotypy (schizoid temperament). Items assess lack of enjoyment from social and physical sources of pleasure and avoidance of intimacy. The ImpNon scale features 10 items generically indexing lack of self-control (impulsive, anti-social, and eccentric forms of behavior). The O-LIFE following its development has become widely used. The scale possesses established psychometric qualities, particularly high internal consistency (Mason et al., 1995) and test–retest reliability (Burch et al., 1998). Since inception, clinical and experimental studies have used the O-LIFE, establishing its legitimacy, reliability and validity (Mason and Claridge, 2006).

#### Revised Paranormal Belief Scale (RPBS) (Tobacyk and Milford, 1983; Tobacyk, 2004)

The RPBS is a 26-item self-report measure, which assesses belief in the paranormal. The RPBS is the most widely used measure of paranormal belief (Goulding and Parker, 2001). Questions are presented as statements (e.g., ‘There is a devil’) and respondents rate items on a seven-point Likert scale, ranging from 1 (strongly disagree) to 7 (strongly agree). Items measure seven facets: traditional religious beliefs, spiritualism, extraordinary life forms, psi, witchcraft, precognition and superstition. The RPBS globally demonstrates satisfactory reliability; sub-scale dimensionality, however, is uncertain (Cardena et al., 2015). To address measurement concerns, Lange et al. (2000) performed a Rasch scaling purification of the scale. This produced two psychometrically superior factors; NAP (11-items assesses belief in psi and survival of bodily death) and TPB (five-items measure belief in concepts, such as the devil, witchcraft, heaven and hell) (Cardena et al., 2015). Rasch scaling demands that responses are recoded (0–6) (Lange et al., 2000). Thus, total scores range from 0 to 156 (higher scores indicating belief in the paranormal). NAP Rasch scores range from 6.85 to 47.72 and TPB 11.16 to 43.24 (Andrich, 1988). Overall, RPBS demonstrates adequate validity (Tobacyk, 2004). Both subscales demonstrate minimal item response bias (gender and age), possess predictive validity and are unidimensional. The RPBS overall, is a psychometrically satisfactory measure of belief in the paranormal (Tobacyk, 2004).

The presentation order of paranormal belief and schizotypy measures was counter-balanced across participants.

### Procedure

Prior to testing ethical approval was granted as part of a research project examining the relationship between anomalous beliefs and cognitive-perceptual measures. Potential participants read an information sheet before consenting to the study. After providing informed consent, participants received the booklet containing the measures. Instructions asked participants to take their time and answer questions as openly and honestly as possible. The booklet contained four sections: personal information (always completed first), problem solving, O-LIFE and belief in the paranormal. On completion of the booklet, participants were debriefed.

### Data Analysis Plan

#### Justification and General Analytical Strategy

Prior to structural equation modeling (SEM) data screening was undertaken. Then means, standard deviations and bivariate correlations for each scale were calculated. SEM characterizes hypothetical constructs as latent variables, which represent interrelated measures or observed variables. Fit indices evaluate the degree to which observed data support specified theoretical models. In the context of the present study, mediation analysis was performed to determine whether level of paranormal belief explained the relationship between schizotypy and propensity to statistical bias (misperception of randomness and conjunction error). Social science research frequently employs mediational analysis because it identifies and elucidates the process that underlies a reported relationship between observed variables. Analysis in the current study used AMOS version 22.

Prior to model evaluation, confirmatory factor analysis ensured sequential estimation of measures was appropriate (Anderson and Gerbing, 1988). SEM included an analysis of general mediation; this assessed the hypothesis that schizotypy had both direct and indirect effects on statistical bias. Particularly, the notion that belief in the paranormal mediated the schizotypy-statistical bias relationship. Mediation was determined by consulting bootstrapping estimates of indirect effects.

Structural equation modeling conventions necessitate a comparison of alternative models, when more than one *a priori* model is available (within the alternative model proposed mediator effects are restricted to zero). The degree to which the less restrictive model better fits the data provides an indication of the significance of the mediator. For comparison purposes, an additional alternative model was tested in which statistical bias was suggested to mediate the relationship between schizotypy and paranormal belief. Finally, consideration of partial correlations indicated whether level of schizotypy associated with endorsement of problems framed in a paranormal context.

#### Fit Indices

Several indices evaluated model fit (the maximum likelihood chi-square statistic, χ^2^; the Root Mean Square Error of Approximation, RMSEA; Standardized Root Mean Square Residual, SRMR; and the Comparative Fit Index, CFI) (Hu and Bentler, 1999). Traditionally, chi-square assesses absolute fit, the degree to which *a priori* model fits or reproduces data. Chi-square, however, is sensitive to sample size and frequently rejects structural models derived from large samples (Kline, 2005). Hence, RMSEA and SRMR are also typically used. RMSEA measures the difference between the population covariance matrix and the reproduced covariance matrix, in order to control for sampling variability. Strength of the RMSEA is that it has a confidence interval (CI), which provides an indication of how precise the fit of a model is. SRMR provides the square root of the discrepancy between the model covariance matrix and the sample covariance matrix.

Relative fit considers relationship between the chi-square from the proposed model with the null/baseline model (i.e., Comparative Fit Index – CFI, Cronbach, 1990). Specifically, CFI compares the chi-square of the hypothesized model with a model that assumes all relationships among measured variables are zero (independence model). CFI values above 0.90 indicate good fit and values above 0.95 specify very good model fit (Hu and Bentler, 1999). In line with previous research (e.g., Bong et al., 2013), CFI values above 0.88 can be considered to indicate marginal fit. An acceptable model requires RMSEA less than 0.10, SRMR less than 0.08 (Browne and Cudeck, 1993), and a CFI greater than 0.88 (Bong et al., 2013). For reporting RMSEA values, the 90% CI was included. Furthermore, Akaike Information Criterion (AIC; Akaike, 1974) evaluated model fit; smaller values indicate better models.

## Results

### Scale Properties and Inter-Measure Correlations

The O-LIFE demonstrated good internal reliability, Cronbach’s alpha (*α* = 0.87). The UnExps (*α* = 0.76) and CogDis (*α* = 0.81) subscales possessed acceptable and good internal reliability respectively. IntAn (*α* = 0.66) and ImpNon (*α* = 0.64) fell below the frequently cited *α* = 0.70 level of acceptability. This was not problematic because 0.60, allowing for measurement error in psychological/social science, represents an acceptable level (Nunnally, 1978; Lance et al., 2006). Observed alpha values were consistent with those reported by Mason et al. (2005). The Revised Paranormal Belief Scale (RPBS) demonstrated excellent internal reliability, Cronbach’s alpha (*α* = 0.94). The NAP (*α* = 0.88) and TPB (*α* = 0.82) subscales possessed good internal reliability (George and Mallery, 2003). Total schizotypy (O-LIFE) and all schizotypy subscales except IntAn indicated significant positive correlations with paranormal beliefs. Of the schizotypy subscales, UnExps possessed the strongest correlations with paranormal beliefs (see **Table 1**).

**Table 1:** Scale descriptive information and inter-scale pearson correlations.

	*α*	*M*	*SD*	1	2	3	4	5	6	7
(1) 0-LIFE	0.87	14.93	7.73							
(2) Unusual experiences	0.76	3.67	2.71	0.79**						
(3) Cognitive disorganization	0.81	5.36	3.18	0.85**	0.53**					
(4) Introvertive Anhedonia	0.66	2.38	2.09	0.56**	0.25**	0.32**				
(5) Impulsive non-conformity	0.64	3.52	2.20	0.78**	0.55**	0.58**	0.25**			
(6) RPBS	0.94	72.44	20.30	0.25**	0.35**	0.19**	-0.03	0.19**		
(7) NAP	0.88	29.30	5.55	0.23**	0.30**	0.18**	0.01	0.18**	0.85**	
(8) TPB	0.82	20.85	5.75	0.23**	0.33**	0.19**	-0.01	0.16**	0.86**	0.72**

O-LIFE, The Oxford-Liverpool Inventory of Feelings and Experiences; RPBS, Revised Paranormal Belief Scale; NAP, New Age Philosophy; TPB, Traditional Paranormal Belief, *p < 0.05, **p < 0.01.

### Problem Type Descriptive Statistics

Problem solution scores were calculated (perception of randomness, conjunction fallacy, paranormal perception of randomness, paranormal conjunction fallacy, overall standard, and overall paranormal). These are presented as means and proportions alongside inter-problem correlations in **Table 2**. Pearson product moment revealed positive correlations between problem types.

**Table 2:** Problem task descriptive information and inter-item pearson correlations.

	*M*	*SD*	Proportion	*SD*	1	2	3	4
(1) Perception of randomness	3.76	1.08	0.75	0.22				
(2) Conjunction fallacy	1.94	1.31	0.39	0.26	0.21**			
(3) Paranormal perception of randomness	4.29	1.10	0.86	0.22	0.39**	0.14*		
(4) Paranormal conjunction fallacy	4.41	1.16	0.88	0.23	0.24**	0.26**	0.51**	

*p < 0.05, **p < 0.01.

### Belief in the Paranormal, Schizotypy and Problem Task Solution

A further set of Pearson product moment correlations found negative associations between belief in the paranormal and schizotypy and problem types (see **Table 3**), particularly UnExps and TPBs.

**Table 3:** Pearson correlations between problem solving task performance, schizotypy and belief in the paranormal.

	O-LIFE	UE	CD	IA	IN	RPBS	NAP	TPB
Perception of randomness	-0.15*	-0.17**	-0.11*	-0.03	-0.12*	-0.25**	-0.17**	-0.28**
Conjunction fallacy	-0.11*	-0.14*	-0.10	0.00	-0.07	-0.17**	-0.10	-0.19**
Paranormal perception of randomness	-0.14*	-0.15**	-0.05	-0.10*	-0.14*	-0.38**	-0.31**	-0.31**
Paranormal conjunction fallacy	-0.24**	-0.32**	-0.15**	-0.11*	-0.13*	-0.49**	-0.40**	-0.40**

O-LIFE, The Oxford-Liverpool Inventory of Feelings and Experiences; UE, Unusual Experiences; CD, Cognitive Disorganization; IA, Introverted Anhedonia, IN, Impulsive Non-conformity, RPBS, Revised Paranormal Belief Scale; NAP, New Age Philosophy; TPB, Traditional Paranormal Belief, *p < 0.05, **p < 0.01.

### Structural Equation Modeling

Examination of inter variable zero-order correlations revealed that belief in the paranormal was most strongly associated with the UnExps factor of schizotypy. Hence, corresponding with predictions, subsequent SEM analyses focused on UnExp. Following confirmatory factor analyses (CFA), SEM assessed the hypothesis that UnExp had both direct and indirect effects on statistical bias. Mediation was determined by consulting bootstrapping estimates of indirect effects and assessed two models: model 1 examined the role of TPB, and model 2 considered the role of NAP. As outlined in the introduction, previous research reports that proneness to statistical bias varies as a function of belief type. Specifically, TPB correlates more strongly with general propensity to statistical bias than NAP.

### Confirmatory Factor Analyses

Analysis involved theoretically driven CFA on each selected scale (UnExp, RPBS standard problem types, and problems couched within a paranormal context). The UnExp subscale, according to research, represents a single factor. The model based on supporting research for the RPBS was a two-factor correlated model consisting of Traditional Paranormal New Age and Philosophy Beliefs factors. Models for standard problem types and paranormal problems were also two-factor correlated models.

The single factor model for UnExps reported a significant chi-square and unsatisfactory model fit for CFI, marginal fit for RMSEA and SRMR: χ^2^(54, *N* = 254) = 157.07, *p* < 0.001; CFI = 0.79; RMSEA = 0.08 (CI of 0.07–0.10); SRMR = 0.07. Further inspection revealed poor factor loadings on the UnExps subscale of items 1 and 9 (0.25 and 0.22 respectively) and so were dropped from the CFA, resulting in a good overall model fit: χ^2^(31, *N* = 254) = 58.91, *p* < 0.05; CFI = 0.94; RMSEA = 0.06 (CI of 0.04–0.08); SRMR = 0.05. Comparison of AIC values supported the superior fit of the factor model following removal of items 1 and 9. The AIC value for the original model was higher (229.07 and 126.91 respectively). For the two-factor model for the RPBS, results showed that although the chi-square was significant, χ^2^(99, *N* = 254) = 345.29, *p* < 0.001, the fit indices met the criteria for a marginal fit: CFI = 0.88; RMSEA = 0.10 (CI of 0.96–0.12); SRMR = 0.07.

The two-factor correlated model for standard problems reported a non-significant chi-square and the fit indices met the criteria for a good fit: χ^2^(34, *N* = 254) = 47.83, *p* > 0.05; CFI = 0.92; RMSEA = 0.04 (CI of 0.01–0.07); SRMR = 0.05. However, scale scrutiny revealed a poor factor loading for problem 16 (0.12). Removing this item from the model resulted in a more parsimonious solution: χ^2^(26, *N* = 254) = 36.51, *p* > 0.05; CFI = 0.94; RMSEA = 0.04 (CI of 0.01–0.07); SRMR = 0.05. Comparison of the AIC values supported the superior fit of the factor model with item 16 removed, as the AIC value of the original model is higher than for the model with item 16 removed (109.84 and 92.52 respectively). Finally, the two-factor correlated model for problems in a paranormal context indicated a significant chi-square, χ^2^(33, *N* = 254) = 95.63, *p* < 0.001, yet all other fit indices met the criteria for acceptable fit: CFI = 0.90; RMSEA = 0.08 (CI of 0.06–0.11); SRMR = 0.05.

Overall, results suggest that the theoretically driven two-factor correlated models satisfactorily represent paranormal beliefs, standard problems and problems in a paranormal context; and that a single factor adequately explains UnExps.

The adequacy of the factor solutions can be also determined in relation to parameter estimates. All factor loadings were positive and statistically significant; all items possessed factor loadings greater than the minimum threshold of 0.32 (Tabachnick and Fidell, 2001). Compliant with the strict factor loading requirements of Hair et al. (1998) the majority of indicators exhibited factor loadings above 0.60.

#### Composite Reliability

Latent modeling cautions that traditional measures of internal reliability (i.e., Cronbach’s *α*) over or underestimate scale reliability (Raykov, 2002). Hence, composite reliability provides a more rigorous assessment of internal reliability. When considering composite reliability, values greater than 0.60 are acceptable (Diamantopoulos and Siguaw, 2000). Results for the standard problems indicated that perception of randomness (PR) (*ρc* = 0.62) and conjunction fallacy (CF) (*ρc* = 0.60) possessed satisfactory composite reliability. Problems framed in a paranormal context, PPR and PCF indicated satisfactory composite reliability (*ρc* = 0.80 and *ρc* = 0.65 respectively), as did UnExp (*ρc* = 0.75). Finally, TPB and NAP, demonstrated also satisfactory composite reliability (*ρc* = 0.78 and *ρc* = 0.88 respectively).

### Model Test: Schizotypy, Paranormal Belief and Standard Problem Types Traditional Paranormal Beliefs

**FIGURE 1:**
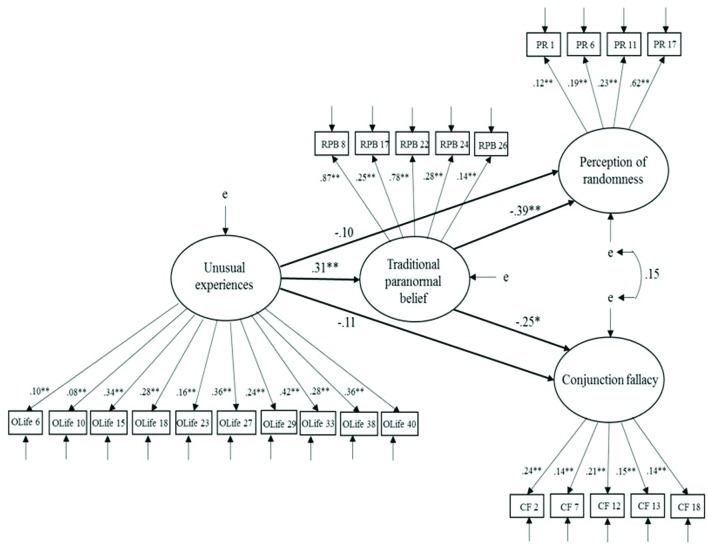
**Model 1 – Mediation model depicting the relationship between unusual experiences and standard problem types, as mediated by traditional paranormal belief.** Latent variables are represented by ellipses, measured variables are represented by squares, and e indicates error of measurement. Lines between latent variables indicate standardized coefficients; *p < 0.05, **p < 0.01.

The mediation model in which TPB subscale had both direct and indirect effects on statistical bias (standard problem types of conjunction fallacy vs. perception of randomness) (**Figure 1**) was statistically significant, χ^2^(239, *N* = 254) = 371.92 *p* < 0.001. Fit indices indicated an acceptable data-model fit: CFI = 0.90; RMSEA = 0.04 (CI of 0.03–0.05); SRMR = 0.07. Inspection of the structural path from UnExp to TPB revealed a significant positive effect of UnExp on TPB (β = 0.31, *p* < 0.001). Furthermore, TPB had a significant negative effect on both perception of randomness (β = -0.39, *p* < 0.001) and conjunction fallacy (β = -0.25, *p* < 0.05). UnExp and TPB accounted for 19% of the variance in perception of randomness, and accounted for 9% of the variance in conjunction fallacy. To formally test whether TPB acted as a mediator, a model was specified where the paths from TPB to UnExp and standard beliefs were constrained to zero. In this model, fit indices indicated an unacceptable model fit on all criteria, but RMSEA, χ^2^(242, *N* = 254) = 418.58, *p* < 0.001, CFI = 0.87, RMSEA = 0.05 (CI of 0.04–0.06), SRMR = 0.11. Referring to the AIC statistic for the mediation model vs. the constrained model for TPB, the mediation model demonstrated superior fit, as the AIC is 541.93, which is lower than the constrained model (AIC = 582.59).

The constrained model indicated a significant negative path between UnExp and PR (β = -0.23, *p* < 0.05), but not between UnExp and CF (β = -0.13, *p* > 0.05). In the final model, however, the path from UnExp to PR was non-significant (β = -0.10, *p* > 0.05). Bootstrapping estimates indicated that UnExp had a lower-bounds indirect effect on PR of -0.25 and upper-bounds indirect effect of -0.07 with *p* < 0.01. This indicated that UnExp had a significant indirect effect on PR. The absence of a significant path between UnExp and CF once TPB was constrained to zero suggests UnExp did not significantly influence CF through TPB. These latter findings suggest that TPB mediated the effect of UnExp on PR, but not CF.

An alternative model was tested in which statistical bias (CF and PR) was proposed to mediate the relationship between UnExp and TPB. Paths between TPB and statistical bias were reversed in this model. Fit indices remained the same, given only direction was adjusted. However, inspection of the paths suggested statistical bias did not fully mediate the relationship between UnExp and TPB, as the path between TPB and UE remained significant (β = 0.20, *p* < 0.05) with the inclusion of statistical bias to this relationship.

### New Age Philosophy

**FIGURE 2:**
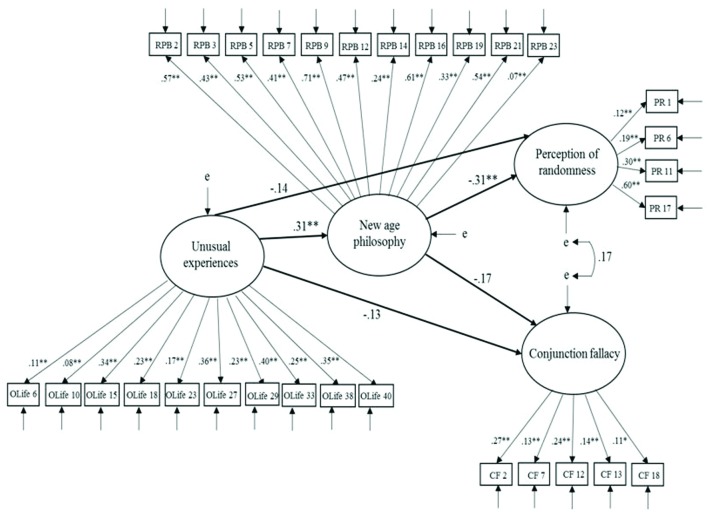
**Model 2 – Mediation model depicting the relationship between unusual experiences and standard problem types, as mediated by new age philosophy.** Latent variables are represented by ellipses, measured variables are represented by squares, and e indicates error of measurement. Lines between latent variables indicate standardized coefficients; *p < 0.05, **p < 0.01.

The mediation model in which NAP had both direct and indirect effects on statistical bias (conjunction fallacy vs. perception of randomness) (**Figure 2**) was statistically significant, χ^2^(392, *N* = 254) = 631.08, *p* < 0.001. Further, consideration of fit indices revealed an acceptable data-model fit: CFI = 0.89; RMSEA = 0.05 (CI of 0.04–0.06); SRMR = 0.07. Examination of the structural path from UnExp to NAP revealed NAP had a significant positive effect (β = 0.31, *p* < 0.001), and a significant negative effect on perception of randomness (β = -0.31, *p* < 0.001) but not on conjunction fallacy (β = -0.17, *p* > 0.05). UnExp and NAP accounted for 15% of the variance in PR, and explained 6% of the variance in CF.

To test formally the mediating role of NAP, the model specified paths from NAP to UnExp and statistical bias were constrained to zero. In this model, fit indices indicated an unacceptable model fit on all criteria, but RMSEA, χ^2^(395, *N* = 254) = 666.33, *p* < 0.001, CFI = 0.88, RMSEA = 0.05 (CI of 0.04–0.06), SRMR = 0.10. Referring to the AIC statistic for the mediation model vs. the constrained model for NAP, the mediation model demonstrated superior fit, as the AIC is 837.08, which is lower than the constrained model (AIC = 866.33). The constrained model indicated that UnExp had a significant negative effect on PR (β = -0.23, *p* < 0.05), but not on CF (β = -0.18, *p* > 0.05). In the final model, however, the path from UnExp to both PR and CF was non-significant (β = -0.14, *p* > 0.05; and *β* = -0.13, *p* > 0.05 respectively). Bootstrapping estimates indicated that UnExp possessed a lower-bounds indirect effect on PR of -0.16 and upper-bounds indirect effect of -0.03 with *p* < 0.05. This indicated that UnExp had a significant indirect effect on PR, and hence NAP mediated the effect of UnExp on perception of randomness. As with model 1, the absence of a significant path between UnExp and CF after constraining NAP to zero suggested that UnExp did not significantly influence CF through NAP.

As with Model 1, an alternative model was tested in which statistical bias (CF and PR) was proposed to mediate the relationship between UnExp and NAP. Paths between NAP and statistical bias were reversed and fit indices remained the same, given only direction was adjusted. Inspection of the paths suggested statistical bias did not fully mediate the relationship between UnExp and NAP, as the path between NAP and UE remained significant (β = 0.22, *p* < 0.05) with the inclusion of statistical bias to this relationship.

### Schizotypy and Paranormal Problem Types

Analysis of paranormal problems omitted belief in the paranormal due to methodological concerns (see Dagnall et al., 2016). Correlating paranormal problems with belief in the paranormal is problematic because it potentially conflates belief and bias. Specifically, problem similarity (shared paranormal context), increases the positive correlation between problem types and reduces bias discriminatory power. Hence, partial correlation identified unique variance between UnExps (schizotypy) and paranormal problem types.

The relationship between paranormal perception of randomness and UnExps (controlling for paranormal conjunction fallacy) was not significant, *r* = -0.01, df = 251, *p* > 0.05. However, a significant positive correlation was observed between for paranormal conjunction fallacy and UnExps (controlling for paranormal perception of randomness), *r* = -0.28, df = 251, *p* < 0.001.

### Conclusion

Paranormal belief factors (TPB and NAP) mediated the relationship between UnExp and perception of randomness, but not between UnExp and conjunction fallacy. TPB demonstrated stronger relationships than NAP with both UnExp and standard problems. Context influenced the relationship between UnExp and statistical bias. Conjunction problems presented in a paranormal context (controlling for perception of randomness) positively correlated with UnExp. Whilst no association between perception of randomness and UnExp (controlling for conjunction) was observed.

## Discussion

As hypothesized, the O-LIFE UnExps subscale was most strongly associated with belief in the paranormal. UnExp moderately positively correlated with paranormal measures (overall paranormal belief; TPB; and NAP). This finding supported previous work delineating a relationship between positive schizotypy (proneness to experiencing strange perceptual-cognitive sensations and magical interpretations) and belief in the paranormal (Hergovich et al., 2008; Dagnall et al., 2010; Dembińska-Krajewska and Rybakowski, 2014).

Whilst UnExp and belief in the paranormal correlated negatively with performance on problem tasks, consideration of zero-order correlations revealed that belief in the paranormal, particularly TPB, was more strongly associated with proneness to statistical bias. As expected, TPB correlated with both perception of randomness and conjunction fallacy, whilst NAP was associated only with perception of randomness. Results aligned with previous work, which reported a stronger relationship between TPB (vs. NAP) and susceptibility to heuristic bias (Wilson, 2013; Dagnall et al., 2014). Overall, findings concurred with the notion that conjunction bias (in this context) represents a specific instance of misrepresentation of chance (Arnott, 1998, 2006; Rogers, 2014; Dagnall et al., 2016).

Paranormal belief factors mediated the relationship between UnExp and perception of randomness, but not UnExp and conjunction fallacy. TPB (vs. NAP) demonstrated stronger relationships with UnExp and standard problems. Particularly, correlations between TPB, UnExp and perception of randomness (compared to conjunction) reflected this. Alternative models (one a constrained model and the other with statistical bias conceptualized as a mediator) were weaker in comparison with the hypothesized model. This provided support for the role of paranormal belief as a mediator.

The presence of mediation supports the postulation that belief in the paranormal acts as a framework for shaping schizotypal related cognitions (odd beliefs and/or magical thinking) (Williams and Irwin, 1991). This interpretation aligns with the notion of a paranormal worldview (Zusne and Jones, 1982). This is a broad perspective that references events to intangible mental and metaphysical processes, rather than observable/physical factors. From this viewpoint, belief in the paranormal represents a coherent, internally logical set of explanations for unusual phenomenon. Within the worldview, proneness to statistical bias is largely attributable to a subjective preferential thinking style, rather than a defective understanding of probability.

The finding that mediation occurred for misrepresentation of chance and not conjunction is with hindsight predictable because proneness to conjunction (vs. perception of randomness) relates less strongly to belief in the paranormal (Dagnall et al., 2014, 2016). Indirectly, this finding provides further support for the notion that conjunction (in this context) represents a specific instance of misrepresentation of chance (Arnott, 1998, 2006; Rogers, 2014). Conjunction indexes less unique variance (as evidenced by weaker effect sizes and inconsistently reported findings). Misperception of randomness accounted for the majority of variance within the UnExp-statistical bias relationship. This was similar to the previously delineated model for belief in the paranormal, however, the relationship between UnExp and statistical bias was weaker (~7% vs. 3% variance).

Within this study, a framing effect occurred; problems couched within a paranormal context proved easier to solve (standard: perception of randomness 75% and conjunction 39% vs. paranormal: perception of randomness 86% and conjunction 88%). This finding was commensurate with previous studies (see; Rogers et al., 2009, 2011; Dagnall et al., 2016). Placing problems in a paranormal context reduces their discriminatory power because framing conflates belief in the paranormal with statistical bias. Thus, it becomes unclear whether believers endorse incorrect solutions because of their level of belief or their susceptibility to statistical error. Accordingly, whilst problems in a paranormal context are easier to solve, this advantage reduces as a function of belief in the paranormal. Indeed, performance on conjunction tasks increased markedly when the problems were located within a paranormal setting (vs. everyday situation).

Framing strengthened the association between UnExp and conjunction fallacy. This may occur because paranormal settings make conjunctions (event co-occurrence) particularly appealing to individuals scoring high on UnExp. Problem structure may suggest/infer a direct causal relationship between constituent elements. A recent paper by Rogers et al. (2016) supports this notion. They found that believers in the paranormal were prone to endorsing conjunctions when a succeeding event confirmed (provided evidence for) the first. For individuals high in UnExp, the paranormal context supports ideations related to magical thinking, unusual beliefs and odd associations. Clearly, this does not apply in the context of paranormal perception of randomness problems, where participants merely estimate the probability of an event/outcome. This suggests that context may affect conjunction susceptibility, whilst perception of randomness is relatively domain general.

Clearly, more work is required in this area. Generally, assessing the degree to which susceptibility to statistical bias varies as a function of context and belief type. Whilst, belief in the paranormal is associated with misperception of randomness, other anomalous beliefs appear more strongly related to proneness to conjunction. Notably, Brotherton and French (2014) found that participants who more strongly (vs. weak) endorsed conspiracy theories made more conjunction errors.

These recent examples suggest that belief type/structure qualifies proneness to statistical bias. In the case of conspiracies, perceived co-occurrence of events is more important than estimation of probability. By their nature, conspiracies are unlikely to be true (Grimes, 2016). Support for conspiracies occurs, when perceived inadequacies in prevailing explanations (A) result in the endorsement of an alternative account (B) (B is dependent on A). This parallels the underlying structure of conjunction error; in order for B to be true A must be false. For example, conspiracists view inaccuracies within the official Roswell, 1947 incident as evidence that an alien spacecraft crashed (Thomas, 1995; Nickell, 2009).

Before concluding, the authors acknowledge that the mediation model used in this paper was cross-sectional. Therefore, findings provide only correlational evidence. Furthermore, scholars report that cross-sectional data sometimes provide biased estimates (Maxwell and Cole, 2007). It is thus important for future research to carry out longitudinal assessments to demonstrate fully a causal relationship from schizotypy to statistical bias through paranormal belief. In relation to this limitation, it is noteworthy that there are theoretical arguments supporting the more primitive status of schizotypal traits over paranormal beliefs. In particular, schizotypy is a trait-like construct with a notable genetic component (see for example Ericson et al., 2011), and schizotypal traits (including UnExp) are less malleable than paranormal beliefs. In addition, paranormal beliefs act as an interpretive framework, which offer structure to schizotypal traits such as UnExp (Williams and Irwin, 1991). From this perspective, it is logical that schizotypal traits influence statistical bias through paranormal beliefs. Weaker observed effects within the alternative model, where statistical bias was conceptualized as a mediator, provided further support for the direction of the hypothesized relationships. Accordingly, the interpretation of the current mediation models is more theoretically plausible than alternatives, which posit schizotypy or statistical bias as mediators.

There are factors, which potentially limit the generalisability of this paper’s findings. These include failure to employ exclusion criteria, educational level and participant gender. With regard to exclusion criteria, paper reviewers identified variables that potentially could influence or confound results (i.e., psychiatric morbidity, substance misuse and exposure to religion). Other similar work has not identified these factors as problematic or employed exclusion criteria (Hergovich et al., 2008; Darwin et al., 2011). Consistent with this approach, which is typical to working with general adult populations, the authors assumed that routine data screening would eliminate extreme outlying data points; control for potentially confounding scores. With regard to consideration of level of education (academic qualifications), no direct measure was necessary. Preceding work reports that statistical bias proneness is a robust phenomenon, largely unaffected by educational variables, such as level of statistical awareness (Tversky and Kahneman, 1983; Fisk, 2004). Moreover, level of education provides only an indirect index of general cognitive ability (McClelland, 1973). Finally, there was no consideration of gender differences; prior research within the area of paranormal belief and statistical bias has failed to either test or report gender differences. Future work may wish to explore whether these factors influence relationships between belief in the paranormal, schizotypy and statistical bias.

Finally, it is important to acknowledge that potential for sample bias exists within paranormal-related studies. Particularly, individuals interested in the paranormal are more inclined to participate in associated research because of the inherent appeal of the subject matter. Subsequently, samples may under represent non-believers scoring high on schizotypy. Accordingly, paranormal believers’ tendency to self-select may produce an overestimation of the relationship between belief and schizotypy. For this reason, caution is required when interpreting results.

## Conclusion

Within clinical groups, there is a well-established relationship between psychosis, cognitive bias and jumping to conclusions (Hassanali et al., 2015). Collectively, evidence suggests that reasoning abnormalities may correlate positively with the formation of unusual beliefs (Lawrence and Peters, 2004). Within the present study, relationships between schizotypy and proneness to statistical bias were weak. Indeed, as proposed by Williams and Irwin (1991), belief in the paranormal played an important mediating effect. Paranormal belief provided a framework for interpreting cognitive-perceptual factors of schizotypy, which resulted in increased susceptibility to misperception of randomness (schizotypy influenced statistical bias through paranormal belief).

## Author Contributions

ND designed the study, lead data collection, supported analysis and was main author. AD lead analysis and was a contributing author. KD assisted the design of the study, co-lead data collection, and contributed to the writing process. AP contributed to the writing process. PC advised on paper and assisted with drafting.

## Conflict of Interest Statement

The authors declare that the research was conducted in the absence of any commercial or financial relationships that could be construed as a potential conflict of interest.
